# Thyroid Ultrasonography Consistently Identifies Goiter in Adults Over the Age of 30 Years Despite a Diminished Response with Aging of the Thyroid Gland to the Effects of Goitrogenesis

**DOI:** 10.1100/tsw.2001.55

**Published:** 2001-07-17

**Authors:** Sheela R. Brahmbhatt, Rajesh M. Brahmbhatt, Creswell J. Eastman, Steven C. Boyages

**Affiliations:** Department of Diabetes and Endocrinology, Westmead Hospital, NSW 2145, Australia

**Keywords:** IDD, goiter, urinary iodine, blood TSH, palpation, thyroid ultrasound, goitrogens, flavonoids

## Abstract

Iodine deficiency is a national health problem in India and we have recently reported on the severity of IDD in adults and children in Gujarat province. The aim of this study was to determine the utility of thyroid ultrasonography to detect goiter in adults from an iodine-deficient population of Gujarat. We studied 472 adults selected by random household surveys. Data were collected on height, body weight, mid-upper arm circumference, thigh circumference, triceps skinfold thickness, thyroid size (palpation and ultrasonography), and diet. Casual urine samples for iodine (UI) and blood spots for TSH estimation were obtained. Endemic goiter is a major public health problem in Gujarat State, India and is probably caused by multiple factors including iodine deficiency, malnutrition, and other dietary goitrogens. These results indicate that thyroid US consistently detects goiter in adults despite a diminished thyroidal response to variable goitrogenic stimuli.

## INTRODUCTION

Iodine deficiency is a major public health problem that presents as goiter and thyroid hormone dysfunction. This dysfunction, if severe, leads to irreversible brain damage, in its extreme forms visible as endemic cretinism[1,2,3,4]. The epidemiological assessment of the severity of iodine deficiency largely rests with determination of urinary iodine status and of thyroid gland size in specified population groups[5]. Thyroid gland size is largely assessed in children, in part because of the easy availability of these subjects, and also because it reduces the confounding effect of duration of iodine deficiency on thyroid gland responsiveness to changes in iodine status[6].

Iodine deficiency is a national health problem in India. We have recently reported on the severity of IDD in adults and children in Gujarat province[7,8,9,10]. We found that the prevalence of IDD in children was extreme and was explained by a combination of factors, including iodine deficiency, coincident ingestion of goitrogens, and malnutrition. Assessment of iodine deficiency is difficult since it combines indirect measures of current iodine intake, urinary iodine, with indirect measures of past iodine intake, specifically thyroid size. The currency of the latter appears to diminish with age, but there has been no definitive study demonstrating the lack of utility of measuring thyroid size in an endemic goiter population. World Health Organization (WHO) and International Council for Control of IDD (ICCIDD) have indicated that goiter rates after the age of 30 years may not be reliable indicators of current iodine intake[5].

## RESULTS AND DISCUSSION

### Goiter Prevalence

#### *Palpation*

Total goiter rate (TGR) by palpation was approximately 10% (45/472) with most of the subjects having grade 0 and 1 goiter according to WHO classification. Goiter grade 2 was seen in two males and five females. TGR by palpation was higher in Dang (31.3%) compared to Baroda (5.1%). Females were more affected (32/227 = 13.7%) as compared to males (13/245 = 5.1%) (Table 1).

#### *Ultrasonography*

Goiter prevalence by ultrasound was found to be much greater, 100% in Dang and 78.6% in Baroda (Table 1). The median thyroid volume for the study group was 36.6 ml (Interquartile Range [IQR]: 23.4-47.1 ml). Enlarged thyroid volume (TV) (as defined greater than 20 ml) was found in 82.2% of subjects (388/472) Table 1. The median thyroid volume was higher in Dang (46.8 ml) compared to Baroda (32.7 ml) ($p$ < 0.001). There was no significant difference in thyroid volumes by gender ($p$ = 0.8).

These findings affirm previous studies that thyroid palpation underestimates the prevalence of goiter[6,9,11,12,13]. It highlights the importance of field thyroid ultrasound as a tool to monitor the effectiveness of iodine prophylaxis programs.

**TABLE 1**
**Thyroid Size as Determined by Palpation (WHO Classification) vs. Ultrasound**

| Thyroid size | | Male n (%) | Female n (%) | Baroda n (%) | Dang n (%) |
|---|---|---|---|---|---|
| *Thyroid palpation* | | | | | |
| | Grade 0 | 232 (94.7) | 195 (86.3) | 372 (94.9) | 55 (68.7) |
| | Grade 1 | 11 (4.5) | 27 (11.5) | 17 (4.3) | 21 (26.3) |
| | Grade 2 | 2 (0.8) | 5 (2.2) | 3 (0.8) | 4 (5.0) |
| Total | (n = 472) | 245 (100) | 227 (100) | 392 (100) | 80 (100) |
| *Thyroid ultrasound* | | | | | |
| TV 1 | (> 20 ml) | 187 (76.3) | 179 (78.9) | 286 (73) | 80 (100) |
| TV 2 | (> 20 ml) | 193 (78.8) | 195 (85.9) | 308 (78.6) | 80 (100) |
| Iodine in water (μg/l) | | | | 32 | 0 |

TV 1: Thyroid volume calculated by WHO formula.
TV 2: Thyroid volume calculated by rotation ellipsoid model formula.

To determine the sensitivity of thyroid ultrasound at different ages we divided the subjects into four groups (Table 2). In addition, the ratio of thyroid volume to body weight (Wt), TV to height (Ht), and TV to body surface area (BSA) was also calculated to determine the impact of anthropometry on goiter prediction. We found that absolute thyroid volume decreased with age up to 70 years. There was a similar decrease found in the ratios of TV to wt and TV to BSA with increasing age. The TV:Wt ratio was higher in males in the age group 30-49 years ($p < 0.001$), whereas the TV:BSA ratio was higher in females in the same age-group ($p < 0.001$). We found no significant gender difference in TV:Ht ratio in age groups over 69 years (Table 2).

**TABLE 2**
**Age and Sex Distribution of Thyroid Volume (TV), Body Weight and Ratio of TV to Weight, Height, and BSA**

| Age (years) | Sex | No. | TV 2 (ml) Mean ± SD | TV:Wt (ml/kg) | TV:Ht (ml/cm) | TV:BSA (ml/m$^2$) | TGR% US |
|---|---|---|---|---|---|---|---|
| 16 – 29 | **F** | 71 | 40.2 ± 26.4 | 1.00 ± 0.65 | 0.26 ± 0.18 | 30.4 ±19.8 | 77 |
| | M | 102 | 41.7 ± 16.3 | 0.91 ± 0.39 | 0.26 ± 0.10 | 28.4 ± 11.5 | 88 |
| 30 – 49 | **F** | 113 | 35.3 ± 16.5 | 0.68 ± 0.32 | 0.25 ± 0.10 | 27.7 ± 11.1 | 85 |
| | M | 102 | 38.1 ± 14.8 | 0.87 ± 0.38 | 0.23 ± 0.10 | 22.7 ± 10.3 | 71 |
| 50 – 69 | **F** | 36 | 31.0 ± 15.3 | 0.64 ± 0.28 | 0.21 ± 0.13 | 22.0 ± 13.5 | 64 |
| | M | 32 | 30.9 ± 20.2 | 0.66 ± 0.41 | 0.19 ± 0.09 | 20.6 ± 9.4 | 63 |
| over 70 | **F** | 7 | 24.6 ± 13.4 | 0.55 ± 0.27 | 0.23 ± 0.10 | 28.4 ± 12.7 | 82 |
| | M | 9 | 34.1 ± 12.6 | 0.94 ± 0.46 | 0.15 ± 0.09 | 16.2 ± 9.3 | 56 |

TV = thyroid volume; BSA= body surface area; TGR = total goiter rate; US = ultrasonography.

These findings indicate that the sensitivity of the thyroid gland to the effects of goitrogenesis diminishes with age. This adaptive response, however, does not diminish the value of goiter detection in adults since the prevalence rate of goiter was unchanged in the various age groups. Since adult females are the most at-risk population for the vertical effects of iodine deficiency on the offspring, presentation of such material to these adults may assist in persuading communities to consider IDD as being an important public health issue. This approach may increase uptake of preventative measures such as consumption of iodized salt.

Further correction of thyroid size for body size revealed that using absolute volumes might underestimate the severity of thyroid enlargement, in particular in malnourished populations. We found that there was stronger correlation of thyroid size to height and to BSA than to weight in this population. Thyroid volume should be preferably corrected for body surface area as other studies have reported in malnourished populations[14].

## Biochemical Status

### *Urinary Iodine (UI)*

The median UI for the study group was 72 µg/l (IQR: 40-117 µg/l). Dang subjects had a lower median UI (40 µg/1, IQR: 19-67 µg/l) compared to the Baroda subjects (80 µg/l, IQR: 50–120 µg/l).

In Dang, 100% of the males and 90% of the females had UI proportions below 100 µg/l, whereas 76% males and 63% females had a UI proportion below 50 µg/l. In Baroda 69% females and 59% males had a UI proportion below 100 µg/l and 30% females and 23% males had a UI proportion below 50 µg/l (Fig. 1).

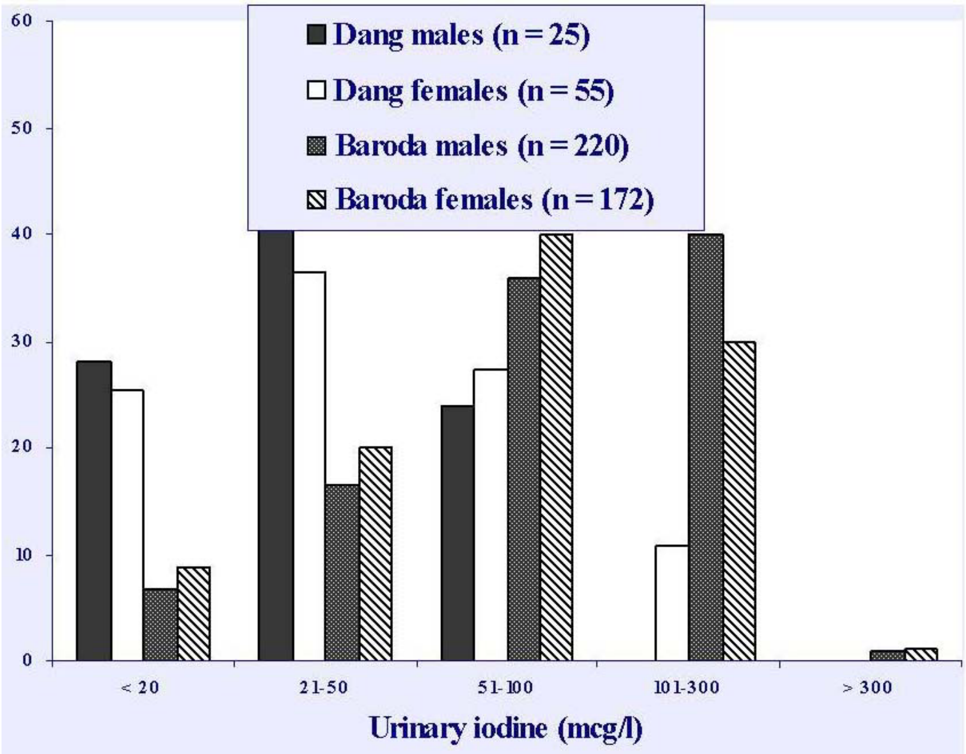

**FIGURE 1.** Frequency distribution of urinary iodine (µg/l) by gender and district in Gujarati adults (n = 472).

In Fig. 1, Y-axis represents percentage of population in each category.

| UI (μg/l) | Iodine status |
|---|---|
| < 20 | Iodine deficiency |
| 21 – 50 | Iodine deficiency |
| 51 – 100 | Iodine deficiency |
| 101–300 | Adequate iodine intake |
| > 300 | More than adequate iodine intake |

Despite the high prevalence of goiter, urinary iodine levels were found to be in the mild to moderate range. Based on these results, the Dang district would be categorized as having moderate iodine deficiency and Baroda as having mild iodine deficiency[7,8]. This would support the argument that goiter in this population is multifactorial in nature, including iodine deficiency, malnutrition, and the ingestion of goitrogenic compounds. These compounds include thiocyanates, goitrin, and flavonoids[9,10].

### *Blood Spot Thyroid-Stimulating Hormone (TSH)*

The study group had a relatively high mean blood TSH level of 1.96 ± 3.2 mU/l. The mean TSH was higher in Dang (2.4 mU/l; SD ± 4.9 mU/l) compared to that in Baroda (1.7 mU/l; SD ± 1.8 mU/l) ($p < 0.01$). There was a statistically significant gender difference in blood TSH levels in Dang ($p = 0.05$), with females more affected than males. Normative values for TSH from an iodine-replete population from India are not currently available. Hence, we have used arbitrary cut-off values of 3 and 5 mU/l for the present study. Whole blood TSH values greater than 5 mU/l were seen in 5% of the adult population. Twenty-four percent of women from Dang had blood spot TSH > 5 mU/l whereas 27% women and 4% men had TSH > 3 mU/l. Three percent each of men and women from Baroda had blood spot TSH > 5 mU/l, whereas 14% women and 8% men had TSH > 3 mU/l (Fig. 2). There was also a relationship between the degree of iodine deficiency and thyroid hormone dysfunction.

The greatest risk of iodine deficiency for a population is its effects on cognitive potential of a community through disturbances of thyroid hormone. The present study showed that TSH values were elevated in this adult population, and more commonly so in females. Almost 10% of women had TSH values greater than 5 mU/l, a value considered to be abnormal in iodine-replete communities. Most of these iodine-deficient women were in the childbearing group and thus likely to pass on their iodine deficiency to their fetuses and infants, leading to irreversible neurologic deficits and brain damage[1,2,3,4]. Normative data for such biochemical measures in iodine-replete communities are urgently needed.

### Relationships

Simple linear regression between thyroid volume and various anthropometric parameters like weight, height, body mass index (BMI), BSA, mid-upper arm circumference (MUAC), triceps skinfold (TSF), and thigh circumference (TC) was performed. This showed a weak ($r = -0.1$) but statistically significant ($p < 0.05$) negative correlation between TV and MUAC (Fig. 3) as well as AMA; in other words, the poorer the protein nutrition the larger the thyroid size. There was no correlation between TV and other anthropometric parameters.

Thyroid volume values were transformed into logarithmic values (LN-TV) to study simple linear regression with other parameters like age, sex, height, weight, BMI, district, UI, TSH, TSF thickness, TC, MUAC, BSA, and consumption of iodized salt. There was a statistically significant

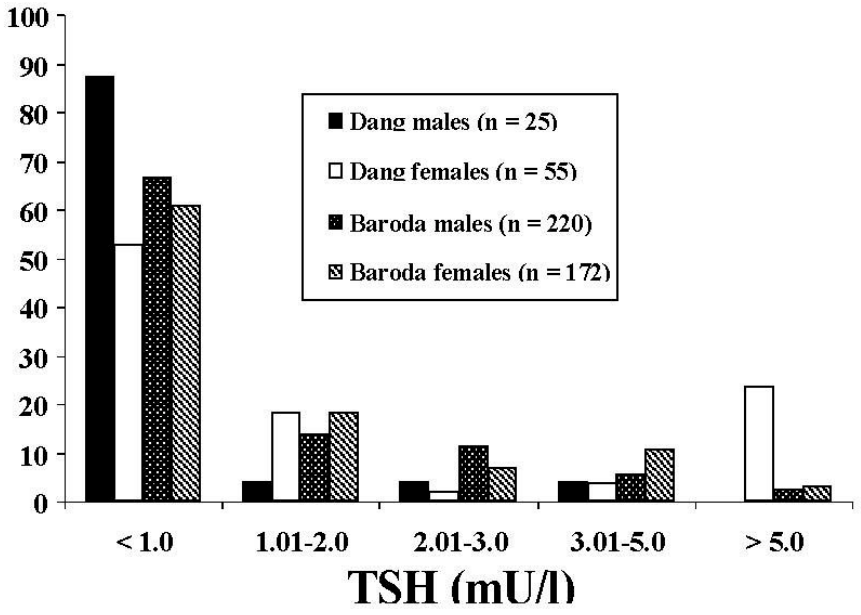

**FIGURE 2.** Frequency distribution of TSH (mU/l) in described ranges by gender in Dang and Baroda. The Y-axis represents the percentage of population.

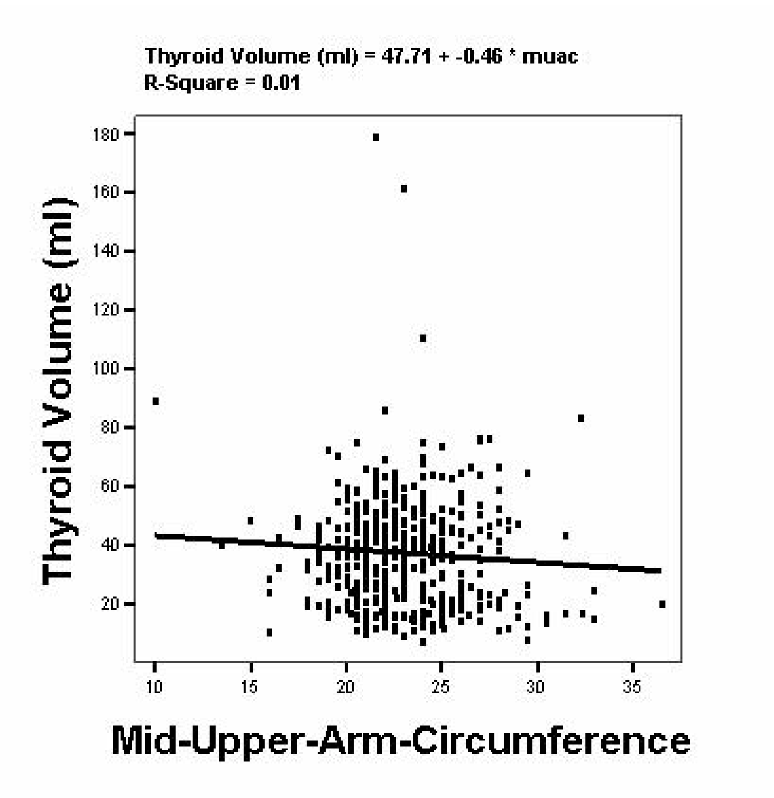

**FIGURE 3.** Linear regression analysis.

($p$ = 0.002) positive correlation between LN-TV and TSH (r = 0.36). A weak but statistically significant ($p < 0.05$) negative correlation existed between LN-TV and age, thigh circumference, MUAC, and iodized salt (r = -0.20, -0.12, -0.12, -0.20, respectively). We did not see any correlation between thyroid volume and body weight.

The best fitting multivariate linear regression model for LN-TV selected using backward elimination accounted for only 20% ($R^2$) of the variability in the LN of thyroid volume. In the adults the significant independent predictors of LN-TV are age, geographic location (district), interfering substances (most probably dietary goitrogens), nutritional status parameters (TSF thickness, TC, and MUAC), and BSA (Table 3).

**TABLE 3**
**Best Model of Linear Regression Analysis Response: LN of Thyroid Volume**

| Parameter | Coefficient | Standard Error | *p* value |
|---|---|---|---|
| Constant | 2.703 | 0.240 | 0.000 |
| Age | -0.005 | 0.002 | 0.003 |
| District | 0.498 | 0.060 | 0.000 |
| Interfering substances | 0.001 | 0.000 | 0.005 |
| Skinfold thickness | 0.021 | 0.006 | 0.000 |
| Thigh circumference | -0.017 | 0.005 | 0.002 |
| Arm circumference | -0.025 | 0.011 | 0.026 |
| BSA | 0.878 | 0.117 | 0.000 |

## SUBJECTS

### Geography

Gujarat is the western most state in India (Fig. 4) with a population of about 42 million spread out in 19 districts. Baroda district is located in the central Gujarat and is well connected to all major cities of India whereas Dang district is an isolated hilly area in south Gujarat. The geography and epidemiology have been previously reported[9].

### Diet

A detailed dietary history was obtained through quantitative and qualitative questionnaires from all subjects. The Gujarati diet is predominantly vegetarian and consists of cereals (pearl millet, jowar, maize, wheat, rice, etc.), beans and legumes (including split-beans like tuver-dal and black udad-whole), and vegetables (cauliflower, cabbage, onion, and tomato with spices, garlic to make a curry, etc.). No other cereal/bean except pearl millet contained flavonoids apigenin, vitexin, and glycosyl-vitexin[9]. The main beverage consumed is tea.

The majority of the subjects consumed noniodized salt because it is cheaper compared to the iodized salt. The most important source of iodine was drinking water with an average daily consumption being two liters due to the hot tropical climate of India.

### Population Studied

The majority of the population was rural (Baroda) and tribal (Dang) and belonged to a low socioeconomic stratum. The population of Muval and Tentalav villages in Baroda district and

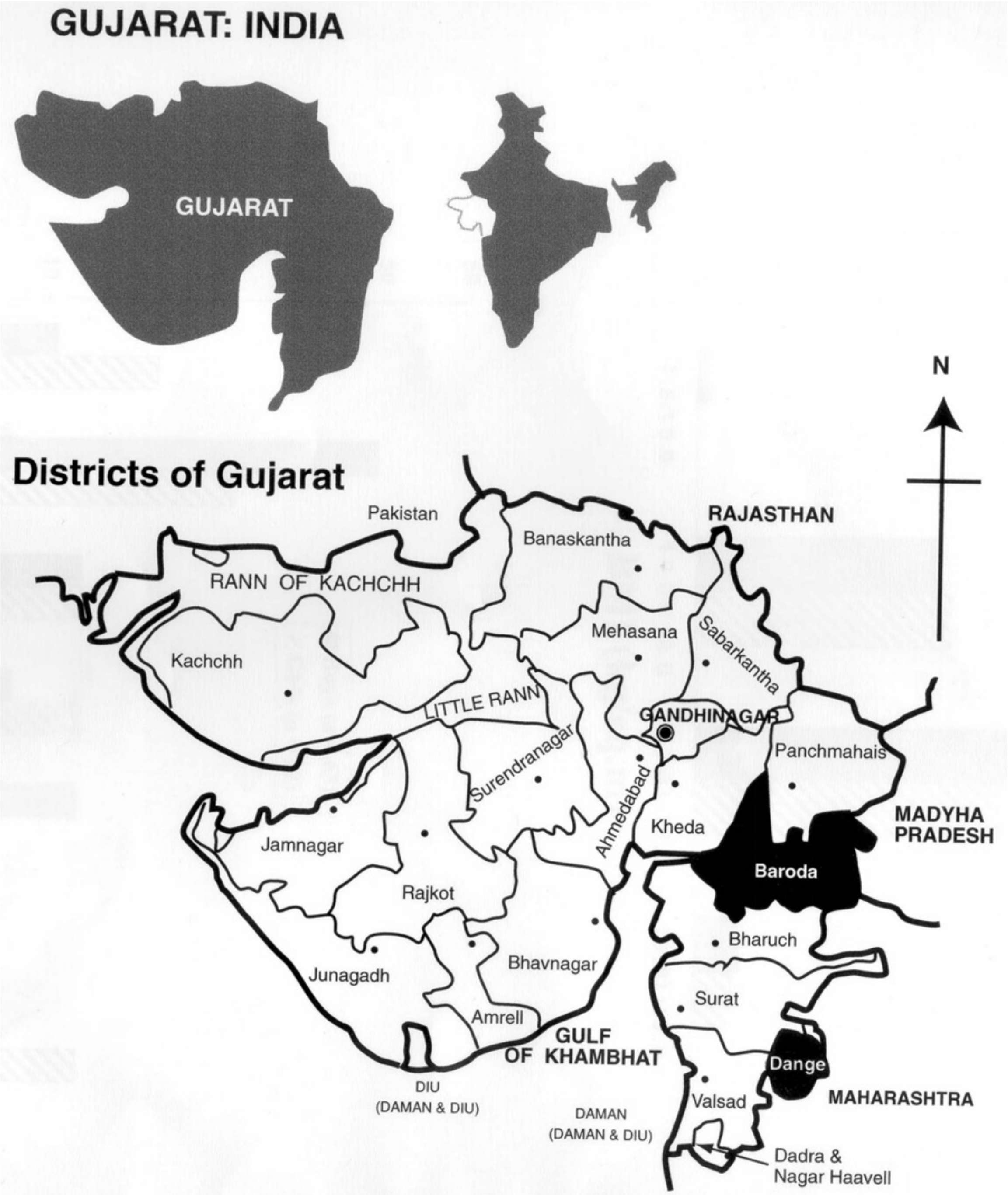

**FIGURE 4.** Map of Gujarat State, shown as a part of India, with the area of study — Baroda and Dang — as shaded districts.

Vaghai, Rambhas, and Baripada villages in Dang district was approximately 70,000. We studied 472 subjects (aged 16 to 83 years; 245 men and 227 women) randomly by carrying out household surveys in Baroda (n = 392) and Dang (n = 80) districts (Table 1).

The subjects were evenly distributed in different age groups and nearly equal numbers of males and females were included with a special emphasis to inclusion of females in the childbearing age group. Fifty-eight percent of men and 69% of women were above the age limit of 30 years. Data on height, weight, TSF thickness, TC, and MUAC was recorded. BSA[15] and BMI were calculated using standard formulae.

Statistically significant gender differences ($p$ = 0.0001) were noted in body weight, height, and BSA in both districts. Baroda males were malnourished as evidenced from BMI ($p$ = 0.0001).

## METHODS

### Thyroid Palpation

The simplified WHO classification[5] for goiter grading was used:

Grade 0: No palpable or visible goiter.
Grade 1: Palpable but not visible.
Grade 2: Visible goiter when the neck is in the normal position.

### Thyroid Ultrasonography

Ultrasound scanning of thyroid gland was carried out as recommended by Brunn et al.[16] using an Ausonics portable ultrasound machine with a standard 5.0 MHz transducer. Longitudinal and transverse scans were performed to measure the thickness, width, and length of each lobe. The volume of each lobe (V) was calculated by two formulae[17,18].

WHO formula:

$$V\ (\text{ml}) = 0.000479 \times \text{Length (mm)} \times \text{Width (mm)} \times \text{Thickness (mm)}$$

Rotation ellipsoid model formula:

$$V\ (\text{ml}) = 0.00052 \times \text{Length (mm)} \times \text{Width (mm)} \times \text{Thickness (mm)}$$

Thyroid volume (TV) was calculated as the sum of the volumes of both lobes. The volume of the isthmus was not included. Thyroid glands were classified as ‘normal’ when the calculated thyroid volume was below 20 ml and ‘enlarged’ when it was more than 20 ml[19]. A single observer (RMB) was used in the determination of TV in this study and the intraobserver variation is usually between 2 and 15%[20].

### Urinary Iodine (UI)

Modified acid digestion method (method E), based on the reaction between cerium IV and arsenic III (Sandell-Kolthoff Reaction) using a Technicon Autoanalyzer II was used[21,22]. The results were expressed as micrograms of iodine per liter of urine (µg/l). This method has several potential sources of error and does not separate out the interfering substances (goitrogens) from iodine, hence the former were removed from the urine samples prior to the assays to arrive at true urinary iodine value[7,8].

### Thyroid-Stimulating Hormone (TSH)

Blood spot TSH levels were measured by commercially available Bioclone neonatal TSH ELISA kits for the quantitative determination of TSH in human neonatal blood spots, manufactured by Bioclone Australia Private Limited, Australia.

### Statistical Methods

Proportion, mean, standard deviation, median, and interquartile ranges have been used to describe the data as appropriate. Linear regression analysis was performed between thyroid volume and anthropometric parameters. Nonparametric tests (Mann-Whitney and *t* test) were performed to study the district and gender differences. Statistical analysis was performed using SPSS version 6.1.2.

## CONCLUSIONS

Goiter was highly endemic in this adult population of Gujarat, India despite many years of public health attempts to eradicate the problem. Goiter prevalence of 82% by ultrasound is very high compared to the degree of iodine deficiency that is mild. This points to a probable multifactorial involvement of iodine deficiency, dietary flavonoids, and malnutrition for the development of goiter. Thyroid palpation was of limited value in adults as grade 0 and 1 goiter predominated, whereas thyroid volume measurement by ultrasound consistently identified goiter in subjects over the age of 30 years despite a diminished response with aging of the thyroid gland to the effect of goitrogenesis. The study confirms our previous findings of other population groups of Gujarat and indicates that urgent action should be taken to implement and monitor prophylaxis programs. This data should be considered in the light of recent government decisions to reverse the ban on the sale of noniodized salt, which can only worsen the situation.
